# Under- and post-graduate training to manage the acutely unwell patient: a scoping review

**DOI:** 10.1186/s12909-023-04119-1

**Published:** 2023-03-03

**Authors:** Helen R. Church, Deborah Murdoch-Eaton, John Sandars

**Affiliations:** 1 Faculty of Medicine and Health Sciences, University of Nottingham, University of Nottingham Medical School, Queen’s Medical Centre, Nottingham, NG7 2UH England, UK; 2 Academic Unit of Medical Education, The University of Sheffield, Sheffield, England, UK; 3 Faculty of Health, Social Care and Medicine, Edge Hill University, Ormskirk, England, UK

**Keywords:** Under-graduate education, Post-graduate education, Acutely unwell patient management, Medical education, Preparedness for practice, Simulation education

## Abstract

**Background:**

Junior doctors are often the first responders to acutely unwell patients and yet frequently report feeling under-prepared to do so. To understand whether this is consequential of how medical students and doctors are trained to manage acutely unwell patients, a scoping review was conducted using a systematic approach.

**Methods:**

The review, informed by the Arksey and O’Malley and PRISMA-ScR guidelines, identified educational interventions targeting the management of acutely unwell adults. Seven major literature databases were searched for journal articles published in English from 2005 to 2022, in addition to the Association of Medical Education in Europe (AMEE) conference proceedings from 2014 to 2022.

**Results:**

Seventy-three articles and abstracts were eligible for the review, the majority of which were from the UK or USA, and demonstrated that educational interventions were more commonly targeted at medical students than qualified doctors. The majority of studies used simulation, but very few integrated complexities of the clinical environment within scenarios such as multidisciplinary working, distraction-handling techniques and other non-technical skills. A wide range of learning objectives pertaining to acute patient management were stated across studies, but few explicitly cited educational theory underpinning their study.

**Conclusions:**

The results of this review encourages future educational initiatives to consider enhancing authenticity within simulation to promote transfer of learning to clinical practice, and use educational theory to augment the sharing of educational approaches within the community of clinical education practice. Additionally, increasing the focus on post-graduate learning, building upon undergraduate educational foundations, is essential to promoting lifelong learning within the ever-changing healthcare environment.

**Supplementary Information:**

The online version contains supplementary material available at 10.1186/s12909-023-04119-1.

## Background

Due to the hierarchical arrangement and delegation of workload within the hospital, it is the most junior members of the medical team that are most frequently called to attend and initiate immediate management for acutely unwell patients [1]. Although the theoretical aspects of acute patient care are taught during undergraduate training, there are internationally-shared concerns from both newly-qualified doctors and their clinical supervisors surrounding preparedness to apply this within the real clinical context [2–4].

Acute care education is challenging. Classroom and simulation-based settings are criticised for failing to replicate stressful environments [5] and genuine clinical encounters are not ideal teaching demonstrations as urgent medical treatment cannot be delayed whilst the nuances of a potentially life-saving interventions are discussed. Therefore, by the time graduates begin their clinical practice, they will encounter aspects of acute care that they have not rehearsed before. Lefroy et al. [6] explored this through junior doctors’ experiences of clinical ‘firsts’. They concluded that although acute patient management can be somewhat prepared for in medical school, undertaking this task alone or being the first attender at a cardiac arrest were situations for which “total prior preparedness is prevented by the step change in responsibility” upon graduation.

Preparation for practice during undergraduate training is often limited by the restrictions on what medical students are ‘allowed’ to do [7], and post-graduation training may be limited since it assumes that doctors are competent from their first day of practice. Smith et al.’s [1] review of undergraduate training in the care of the acutely ill patient reported that training was “sub-optimal, adding to patient risk”. Since [6] Smith et al.’s review in 2007 the clinical environment has become more complex due to a population with increasing age [8], multi-comorbidity [9] and polypharmacy. Arguably too, expectations surrounding healthcare provision have also increased with advancing technologies and therapeutics. How have medical and clinical educators addressed these issues to ensure our most junior doctors are able to provide this care? And how has this changed since the previous review? To understand the current approaches in both under- and post-graduate training we undertook a scoping literature review. A scoping review allows rapid collection and dissemination of current evidence on a research topic [10] and encourages both quantitative and qualitative data to be considered [11].

The purpose of this literature review is to present the current strategies in medical education used to teach medical students and junior doctors how to manage the acutely the unwell patient. The most recent similar review was published over 10 years ago [1]. This review additionally aims to identify gaps in current training strategies and highlight new areas for innovation to better equip the healthcare workforce of the future in maintaining patient safety.

## Methods

**Fig. 1:**
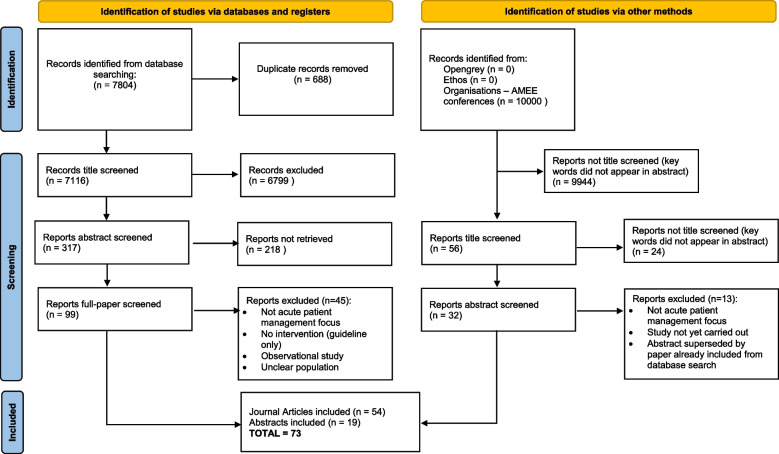
PRISMA [12] flow chart demonstrating outcomes of search process. *From: * Page MJ, McKenzie JE, Bossuyt PM, Boutron I, Hoffmann TC, Mulrow CD, et al. The PRISMA 2020 statement: an updated guideline for reporting systematic reviews. BMJ 2021;372:n71. doi: 10.1136/bmj.n71. For more information, visit: http://www.prisma-statement.org/

To uphold the values of a rigorous scoping review, the Arksey and O’Malley [10], 5-stage framework was adhered to. Figure 1 demonstrates this process in the Preferred Reporting Items for Systematic Reviews and Meta-Analyses (PRISMA) [12] flowchart format.

### Stage 1: identifying the research question

The research questions to be answered by this literature review are:

1. What types of interventions have been used to teach medical students and junior doctors regarding management of the acutely unwell adult patient?
2. Are these interventions more frequently targeted at medical students or junior doctors?
3. What are the underlying educational theories behind the interventions?
4. Do any interventions offer strategies to manage the complexities of the real-life clinical environment? The “acutely unwell adult patient” is defined as a person over 16 years of age who is experiencing an acute medical or surgical emergency

### Stage 2: identifying relevant studies

Seven widely used literature databases were systematically searched (Web of Science, Medline, PubMed, PsychInfo, ERIC, Open Grey and the British Library e-thesis online service). Multiple search terms based on 5 key domains; ‘acutely unwell’, ‘management’, ‘doctor/medical student’, ‘education’ and ‘patient’ were generated iteratively with support from an information librarian (For full details see Additional file 1). Journal articles published between 01/01/2005 and 21/11/2022 were selected for inclusion. A start year for the review of 2005 was chosen since Smith et al’s [1]. review on the topic included studies published to this date; additionally the focus was on studies which were considered to be more relevant to current educational practice and the context of healthcare. The initial search included medical students and doctors of any stage/grade to ease retrieval but only interventions involving medical students and doctors in training were included in this review.

Conference abstracts were identified through electronically searching AMEE conference proceedings using the key words “acutely” and “unwell”. A search range of 2014-2022 was chosen based upon the study outcomes of Walsh et al. [13], who demonstrated a median time of 20 months between medical education research abstract presentation and subsequent publication in a peer-reviewed journal. Furthermore, more than 90% of these abstracts were published within 4 years. Therefore, we anticipated that high-quality abstracts featured in conferences prior to 2014 would have been further developed and published into full journal articles.

Only journal articles and abstracts published in English were included to avoid translation error.

### Stage 3: selecting the studies

Exclusion criteria included interventions in the five clinical specialties shown in Additional file 2. Remaining articles were screened sequentially by title, abstract and full-text.

Identified abstracts were read and selected using the same specialty and target population exclusion criteria as used for the journal search (Additional file 2). A 20% random sample of the reports and abstracts was reviewed by another researcher (JS). Any uncertainties regarding article inclusion were discussed until a consensus was achieved.

### Stage 4: charting the data

Data extraction was guided by Armstrong et al.’s [11] identification of themes during a scoping review and adapted TREND (Transparent Reporting of Evaluations with Non-randomised Designs) guidelines [14].

### Stage 5: collating, summarising and reporting the results

Data was collated on a Microsoft Excel spreadsheet, (Additional file 3) and the variables pertinent to each theme were extracted from each journal/abstract. The summarised results of these themes are reported below.

At Stages 3, 4 and 5 of the methodology above, initial data selection/extraction was initially undertaken by one author (HC) before a 20% random sample of the selected/extracted data was reviewed by another researcher (JS). Any uncertainties were discussed until a consensus was achieved.

Each of the four outcomes to be addressed by this literature review utilised different data from the selected studies. Much of this was simple to extract, such as year of publication and target population. However, more in-depth analysis was required to identify and classify the underlying educational theories behind the interventions described in the articles. The method used for this was described by Cook et al. [15], who characterised medical education studies as Descriptive, Justification or Clarification. Descriptive research includes a recollection of the events of the research and makes no comparison to another group (e.g. control group) nor states a theoretical basis for the research. Justification studies include comparisons to address whether one intervention is more successful than another. Clarification studies are considered to be more complete in addressing both the aforementioned alongside and state the underpinning theories of the intervention.

## Results

The literature search identified 54 journal articles [16–69] published in the past 17 years and 19 abstracts [70–88] from conferences held in the past 8 years. The results presented below include data from all 73 articles/abstracts unless stated otherwise.

### Who?: target population and number of participants

Table 1 demonstrates that the majority of interventions were aimed at medical students either exclusively (36, 49%) or collaboratively with other healthcare professionals (2, 3%), compared to doctors. However, of the reports which included multidisciplinary team (MDT) studies, doctors were more often participants (*n* = 11, 91% of MDT studies) compared to medical students (*n* = 2, 17%); One study included both doctors and medical students, alongside allied healthcare professionals. Twenty-nine studies (40%) specifically targeted either final-year students or first-year doctors.

**Table 1:** Summary of the characteristics of the identified journals and articles (*n* = 50)

***Target Population***	**Frequency (% of all studies)**		
	**Single professional participants**	**Multi-professional participants**	**TOTAL**
Medical Students	36 (49)	1 (1)	**38 (52%)**
Doctors	27 (37)	7 (10)	**33 (45%)**
Medical Students AND Doctors	1 (1)	1 (1)	**2 (3%)**
***Simulation Fidelity***		**Frequency (% of simulation studies)**	
Mannikin only		21 (38)	
Both Patient Simulator and Mannikin		5 (9)	
Patient simulator		4 (7)	
Task trainer		3 (5)	
Virtual Patient/ Virtual Reality		2 (4)	
Healthcare professional simulator		1 (2)	
Both task trainer and live animals		1 (2)	
Instant Messaging		1 (2)	
Not described		17 (31)	
**TOTAL**		**55 (100)**	
***Country***		**Frequency (% of all studies)**	
UK		32 (44)	
USA		20 (27)	
Australia		3 (4)	
Germany		2 (3)	
Singapore		2 (3)	
India		2 (3)	
Collaboration:			
• Uganda and UK		1 (1)	
• UK and USA		1 (1)	
Canada		1 (1)	
Denmark		1 (1)	
Egypt		1 (1)	
Hong Kong		1 (1)	
Iran		1 (1)	
Jordan		1 (1)	
Malta		1 (1)	
Netherlands		1 (1)	
Sri Lanka		1 (1)	
Thailand		1 (1)	
***Time between intervention and data collection***		**Frequency (% of all studies)**	
Immediately post-intervention (only)		19 (26)	
Immediately and followed-up:			
• Immediately AND within 1 month post-intervention		1 (1)	
• Immediately AND 1-4 months post-intervention		7 (10)	
• Immediately AND 5-8 months post-intervention		1(1)	
• Immediately AND unclear end date		1(1)	
Within 1 month post-intervention		5 (7)	
1- 4 months post-intervention		4 (5)	
5 - 8 months post-intervention		1 (1)	
9 - 12 months post-intervention		2 (3)	
Not clearly defined (e.g., ‘end of semester’)		5 (7)	
Not stated		27 (37)	
**Educational objectives of interventions (Many studies stated more than one educational outcome)**		**Frequency (% of all studies)**	
Confidence/preparedness in assessing/managing acutely unwell patient		46 (63)	
Course evaluation as a learning event		25 (34)	
Observed knowledge-based improvement		29 (40)	
Perceived skills/knowledge gained		5 (7)	
Communication around acutely unwell patient		4 (5)	
Educational motivation/sustained learning		4 (5)	
Course evaluation as an enjoyable event		4 (5)	
Confidence in practical skills		2 (3)	
Patient care outcome		2 (3)	
Curriculum development		1 (1)	
***Year of Publication***		**Frequency (% of journal articles)**	
2005		2 (4)	
2006		2 (4)	
2007		3 (6)	
2008		2 (4)	
2009		1 (2)	
2010		1 (2)	
2011		1 (2)	
2012		4 (7)	
2013		1 (2)	
2014		3 (6)	
2015		8 (15)	
2016		2 (4)	
2017		6 (11)	
2018		3 (6)	
2019		2 (4)	
2020		4 (7)	
2021		6 (11)	
2022		3 (6)	
**TOTAL**		**54 (100)**	

Participant numbers in each intervention ranged from six [71] to 357 [67]. Eight articles/abstracts (11%) did not explicitly state actual numbers of participants involved in their studies, but many instead indicated their scale (e.g., the entire year group took part).

Some authors reported large recruitment numbers but subsequently achieved low retention rates at the conclusion of their study. Of the 357 doctors invited to take part in Xu et al.’s [67] study, 319 completed the pre-intervention questionnaire but only 138 completed post-intervention questionnaires. Conversely, the conference abstract by Rajani [80], only included 17 junior doctors but achieved a 100% follow-up response rate.

### What?: types of intervention

1. Descriptive, Justification, Clarification

As described in Methods, Cook et al.’s [15] classification was used to categorise studies as Descriptive (simply stating outcomes), Justification (comparing interventions or using pre/post-intervention outcomes) or Clarification (theoretically-embedded studies). Twenty-two (30%) articles/abstract met this Clarification criteria, compared with 30 (41%) justification studies and 21 (29%) Descriptive studies.

2. Educational approach

Experiential learning was cited in six of the 55 studies in this literature review which utilised simulation [17, 38, 51, 53, 64, 66] . Woods et al. [85], Cash et al. [30] and Thompson et al. [83] all used near-peer learning as an educational concept, whereby the teaching faculty are only slightly more senior than the students being taught, e.g. newly-qualified doctors teaching final-year medical students. Four papers stated multiple theories behind their educational interventions. For example Phillips et al. [79]cited interprofessional education and scaffolding, Wright et al. [69] cited adult learning, contextualised theory and reflective practice, whilst Fuhrmann et al. [38] cited experiential and adult learning.

3. Simulation

Simulation was used in 41 of the 54 full journal articles and 14 of the 19 conference abstracts from this literature review.

Twenty-one studies (38%) utilised only simulation manikins of varying fidelity. Four studies used a simulated patient (or actor) and a further five used both manikins and simulated patients. One study used both task trainers and live domesticated pigs during their surgical residents preparatory course [25]. During the COVID_19 pandemic, alternatives to face-to-face simulation included instant messaging [66] virtual reality [50] as an alternative to in-person simulation. Seventeen (31%) studies did not specify the fidelity of their simulation equipment.

Of the remaining 18 studies which did not use simulation, educational modalities included classroom settings, immersion in the clinical environment, computer based e-learning and web-based learning platforms.

4. Data type and methods

Sixty-six studies used self-report data measurements, the majority reporting confidence or perceived knowledge acquisition using Likert scales and questionnaires. Seven studies reported only objective data in the form of performance observation (e.g. OSCE). Twenty-seven studies include both self-report and observed data.

### Where?: geographical spread of published studies

Thirty-two (44%) journal papers/abstracts originated from the United Kingdom, 20 (27%) were from the USA and three (4%) from Australia. There were five studies from other European countries excluding the UK. Seven studies were from Asia. Two studies described collaborative work from authors based in different continents, both of which included the UK [19, 26].

### When?: timelines publication and data collection post-intervention

Table 1 demonstrates the spread of publication of the journal articles in relation to their year of publication (given the shorter timeline search strategy for conference abstracts, these were not included in the table to avoid skewing the data). Peaks in publication of journal articles pertaining to acute patient management in 2015, 2017 and 2021, the latter being post-peak of the COVID-19 pandemic.

Nineteen studies only measured their outcomes immediately post-intervention, whereas an additional 13 also collected data several months later. Five studies collected data within a month of the educational intervention, whilst four studies collected data between 1 and 4 months post-intervention. Four studies collected data between 5 and 12 months. Five studies did not clearly define their data collection timeline, using more generic phrases such as ‘at the end of placement’ or ‘end of the year’, whilst 27 studies did not indicate a time-span for data collection at all.

### Why?: study aims

As Table 1 demonstrates, the studies targeted a range of educational outcomes pertaining to acute care management. The most common outcomes were subjective; confidence or preparedness to manage the acutely unwell patient (41 studies, 56% of total), or evaluation of the course as a learning event (25, 34%). Twenty-nine (40%) studies measured observed knowledge improvements. Very few studies seemed concerned with practical skills or more direct patient outcomes, such as time to be given antibiotics in patients with suspected sepsis [39].

## Discussion

This scoping review describes the published work regarding training interventions for medical students and junior doctors in managing the acutely unwell patient.

### Question 1: what types of intervention have been used to teach medical students and doctors about management of the acutely unwell patient?

Simulation is a popular approach for teaching the management of the acutely unwell patient. Although Smith et al.’s [1] review included only a small number of studies that used simulation, they predicted the growing use of simulation to teach acute care to undergraduates. Twelve years later, simulation now plays a dominant role in the teaching strategies of this area and this review highlights the popularity and breadth of application of this learning tool within this context [89]. Simulation offers the opportunity for learners to experience a scenario which is similar to a real-life event but without a threat to patient safety [90]. Importantly, simulation is an effective teaching approach since it provides a structured learning experience with debriefing and feedback on performance.

Objective measurements allow knowledge acquisition or behavioural change to be demonstrated, and therein lies the key to transferability to practice, as outlined by McGaghie et al. (2010) regarding best practice in simulation. Furthermore, there is a recognised disparity between self-assessment and objective ability [91] and therefore use of both subjective and objective data enhances the strength of the outcome measurement [92].

A large proportion of the medical education interventions identified in this review are descriptive [15], and often use only student feedback or self-assessment rather than objective measurements of learning outcomes. Less than one third of the studies in this review collected both subjective and objective data. The majority of studies also included a short time-period between intervention and outcome measurement. This potentially introduces a test re-test bias [93] where short-term knowledge is transferred from pre- to post-intervention, and any long-term knowledge is not tested for. Using immediate post-intervention data collection does not adequately provide an outcome measure of its transferability into the clinical context nor retention of knowledge over time, which is the optimum outcome for most medical educational interventions [94].

### Question 2: are these interventions more frequently targeted at medical students or doctors?

The majority of studies targeted medical students rather than junior doctors. However, 40% of the studies included final-year medical students or first-year junior doctors. This transition period seems very popular for acute patient management and perhaps illustrates the shift in focus towards to preparedness for practice as qualified doctors. Very few interventions had a multidisciplinary approach despite the importance of non-technical skills such as teamwork, leadership and communication during acute patient management [19].

### Question 3: what are the underlying educational approaches behind the interventions?

The theoretical underpinning of studies is not well established in this area of medical educational research. Two explanations for this are the lack of understanding of the theories within medical education and a lack of expectation to state the theory [95]. However, the word-count limitation of publications and extracts can be a challenge for the completeness of reporting, particularly with regard to clarification studies [95]. However, 22 reports, including four conference abstracts, demonstrated the ability to succinctly communicate the theoretical stance underpinning their intervention description.

The importance of theory in the design, implementation and evaluation of educational approaches is that it can clarify ‘how’ and ‘why’ the approach is intended to produce a learning outcome [96]. This clarification provides a greater understanding of the process that has been implemented during the learning approach and increases the opportunity for the transferability of the educational approach to other settings. Despite experiential learning being the cornerstone of simulation, only three of the 46 simulation-based studies explicitly stated this theory. The majority of studies identified in this literature review used a justification-style, with a focus only on the outcome (“did it work”) [15]. However, there has been a progressive increase in clarification studies since 2013 and a similar decline in descriptive studies that focus on only “what was done”. This may signal a change in culture and academic expectation to explain ‘how’ and ‘why’ a successful intervention has been achieved, with particular reference to the underpinning theories underpinning [15].

Word-count limitations can be a challenge in medical education publications and conference abstracts to ensure completeness of reporting [95], particularly with regard to clarification studies [95]. However, 22 reports, including four conference abstracts, demonstrated the ability to succinctly communicate the theoretical stance underpinning their intervention description.

### Question 4: do any interventions offer strategies to manage the complexities of the real-life clinical environment?

Rajani et al. [80], utilised authentic clinical experience on the wards in an attempt to increase preparedness for the complex environment of clinical practice, but neither specifically taught mechanisms for dealing with these complexities. Instead, their interventions relied on deliberate practice and experiential learning to achieve better management of the acutely unwell patient. Similarly, Hoi et al. [76] described in their simulation-based study how the participants had to persevere with acute management skill acquisition by re-attempting the task in the face of failure. They commented that this better represented the realism of patient care, where individual failed tasks within a more complex simulation might be overlooked due to time-pressures or being viewed as lacking priority in the grander scheme of the scenario. However, despite being given the time to re-attempt the skill or task, no specific strategies to better cope with the undertaking of clinical skills within a pressured environment were offered.

In the wider preparedness for practice literature there is evidence of a need to teach more generalised skills to cope with the complexity of the clinical environment. One such study by Thomas et al. [97] aimed to impart distraction management techniques to medical students to allow better focus and task management during busy clinical situations. Similarly, one study aimed to enhance acute patient management through controling the negative emotions that doctors experience in the workplace during stressful clinical situations [33]. Although this study did not measure objective outcomes of clinical performance, it does demonstrate an adjunct to current knowledge-based skill sessions in acute care education.

Very few articles or abstracts incorporated the clinical environment into their studies. Without efforts to address transition to practice, studies risk being a purely academic exercise, potentially limiting their clinical applicability and value in the eyes of the participants.

The COVID-19 pandemic has likely had multiple effects on the educational efforts to teach medical students and doctors how to manage acutely unwell patients. Although the pandemic will have brough an increased urgency to train the current and future workforce [98], the logistics of delivering this educational content was challenged by work-load and avoidance of face-to-face educational delivery. This literature review demonstrates a small number of innovations which circumvent the use of more standardised ‘mannikin-based’ simulation during this period – including instant messaging [66] and virtual reality [50]. Interestingly, the number of journal article publications increased in 2021 (post-peak pandemic); this may indicate the beginning of a wave of other innovations to be shared with the medical education community in the months following this review.

## Strengths and limitations of the review

This review provides a broad and useful assessment of the published studies to guide medical educators in the future design, implementation and research of teaching interventions for managing the acutely unwell patient for medical students and doctors According to Vivekananda-Schmidt and Sandars [99] a scoping review, compared to a systematic review, considers both a wider range of evidence and qualitative and quantitative outcomes in equal weighting. This allows a more complete overview of the literature in this area to address not only ‘what’ or ‘who’ are taught, but equally importantly ‘how’ they are taught.

A systematic process based on the Preferred Reporting Items for Systematic Reviews and Meta-Analyses for Scoping Reviews (PRISMA-ScR) checklist [100] (see Additional file 4). was conducted for this review. Expansion and optimization of the initial search terms through iteration during the dynamic process of literature searching was conducted, along with discussions with an information librarian. In addition, seven well-established databases were utilised an attempt to have a maximum yield of the appropriate literature, ensuring that the same (or as similar as possible) search terms were used consistently across each platform. When extracting and collating data from journals and abstracts, a constant comparison [101] approach to ensure that similar themes were either combined or divided appropriately to best represent the data. During the selection of screened studies and also the data extraction and analysis, there was a 20% audit with discussion between two members of the research team (HRC and JS) until consensus was reached.

Despite approaching this scoping review in a systematic way, this was not a *‘Systematic Review’* and therefore despite these efforts to maximize the breadth of the literature search, it is possible that some studies were overlooked. Also, since only articles describing interventions were included in this review, other reports with interesting but as yet untested guides for educational programmes were exempt due to a lack of data. Despite not excluding healthcare professional search terms, exclusion of keywords pertaining to clinical specialities, e.g. palliative, could also have inadvertently excluded some specialty-overlapping studies which may have been of interest. Similarly, the selection of articles only written in English accounted for approximately 200 articles being excluded prior to title screening. The majority of these articles were written in other European languages such as French and German. On balance, the authors felt that excluding articles not written (or formally translated) in English was preferable to incurring translation error.

## Conclusions

Managing the acutely unwell patient can be very challenging for junior doctors due to the balance between replicating realism and responsibility with urgent patient care. This global problem has been approached in many ways over the past 17 years, but gaps still remain which should be the focus of future research and innovation in this area of medical education It seems logical that acute care education should include strategies to cope with the uncertainties [102] and added complexities in the real-life context of work [103].

This review demonstrates that the majority of interventions in the area of acute care are aimed at medical students. Although this has satisfied the need for more undergraduate-focussed acute care education [1] educational interventions after the first post-graduate year appear to be lacking. Post-graduate education is often difficult to organise as it competes with junior doctors’ clinical commitments. In addition, one might assume that once working, junior doctors gain adequate learning and maintain their skills simply through clinical encounters. This is counter-argued by those who perceive that clinical experience is limited due to restrictions instigated by legislation on junior doctors’workload, such as the European Working Time Directive [104, 105].

Simulation is considered an educational approach which supports transition of learning to practice. However, the studies in this review which used simulation generally failed to capitalise on its potential. Likewise, realism appeared to be limited to the use of high-fidelity manikins, which although considers authenticity from an equipment perspective, fails to acknowledge the importance of environmental and perhaps psychological fidelity on learning [106], in [107].

The COVID-19 pandemic has arguably driven many technological advances, some of which have altered personal and professional daily activities irreversibly. Given the heavy utilisation of digital learning aids (particularly simulation) which are used in the field of medical education pertaining to acutely unwell patients, it is likely that there are more innovations to be shared with the community, some of which might herald a new era of educational innovation.

Future researchers and educators must consider the complexity of the clinical environment when preparing medical students and doctors to deliver optimum acute patient care. By replicating the “messy clinical environment” [108] through creating distractions, involving multidisciplinary team members and embedding non-technical skills, more authentic educational experiences can be created to encourage transfer to real clinical practice.

## Supplementary Information

**Additional file 1.** Phase 1 Search Terms and Number of articles yielded from initial search.

**Additional file 2.** Exclusion keywords for journal article literature search.

**Additional file 3.** Raw Data Set from Scoping Review.

**Additional file 4.** Preferred Reporting Items for Systematic reviews and Meta-Analyses extension for Scoping Reviews (PRISMA-ScR) Checklist.

## Data Availability

All data generated or analysed during this study are included in this published article (and its supplementary information files).

## Abbreviations

PRISMA-SCR: Preferred Reporting Items for Systematic Reviews and Meta-Analyses for Scoping Reviews
MDT: Multidisciplinary Team
OSCE: Observed Structured Clinical Examination
