# Supplementary material for: Under- and post-graduate training to manage the acutely unwell patient: a scoping review
Source: BMC Med Educ. 2023 Mar 3;23:146. doi: 10.1186/s12909-023-04119-1 (PMC9983517; doi:10.1186/s12909-023-04119-1)
Supplement: Supplementary file 1 — Additional file 1. Phase 1 Search Terms and Number of articles yielded from initial search. [file 12909_2023_4119_MOESM1_ESM.docx]

Additional file 1: Phase 1: Search Terms and Number of articles yielded from initial search

For each search database listed, Topics 1 through 5 were combined using the term “AND”

Where topic boxes are left blank adding search terms reduced the yield of papers significantly and were not employed.

“ ” denotes exact phrase search

* allows for truncation searching whereby different endings of a word are searched

exp = “explodes” controlled vocabulary term

.mp = combined search fields (default if no fields are specified)

‘discipline’: where a topic for thesis is chosen to concentrate field of study

| **Database** | **Keywords** | | | | |
| --- | --- | --- | --- | --- | --- |
|  | **Topic 1:**  **Acute patient scenario** | **Topic 2:**  **Patient management** | **Topic 3:**  **Medical doctor** | **Topic 4: Educational intervention** | **Topic 5:**  **Patient care** |
| **Web of Science** | "acutely unwell" OR "acutely ill" OR "deteriorating " OR "acute" OR  "prepared for practice" OR "preparedness" | "manage" OR "management" | "doctor" OR "medic" OR "medical student"  OR "medical" | train* OR  teach* OR "education" | "patient" OR "patients" |
| **Medline** | "acutely unwell" OR "acutely ill" OR "deteriorating" OR "acute" OR  "prepared for practice" OR "preparedness" | "manage" OR "management” | “doctor" OR "medic" OR "medical student" OR "medical" | train* OR  teach* OR "education" | "patient" OR "patients" |
| **Pubmed** | "acutely unwell" OR "acutely ill" OR "deteriorating" OR "acute" OR  "prepared for practice" OR "preparedness" | "manage" OR "management" | "doctor" OR "medic" OR "medical student" OR "medical" | MeSH Term medical education | "patient" OR "patients" |
| **PsycInfo** | "acutely unwell" OR "acutely ill" OR "deteriorating" OR "acute" OR  "prepared for practice" OR "preparedness" | "manage" OR "management" | "doctor" OR "medic" OR "medical student" OR "medical" | medical education.mp OR exp. Medical Education  **AND**  train* OR  teach* OR "education" | "patient" OR "patients" |
| **ERIC** | acute* OR "acutely ill" OR deteriorating  OR "acutely unwell" | management OR manage | postgraduates OR doctor OR foundation | education OR "medical education" OR teach* OR  learn* OR  train* OR develop* OR strateg* | medical |
| **Open Grey** | acute* | manage* | train* OR teach* OR "education* |  | discipline: Medicine |
| **British library e-thesis online service (EThOS)** | acute OR  prepared |  | doctor |  |  |
