# Supplementary material for: Under- and post-graduate training to manage the acutely unwell patient: a scoping review
Source: BMC Med Educ. 2023 Mar 3;23:146. doi: 10.1186/s12909-023-04119-1 (PMC9983517; doi:10.1186/s12909-023-04119-1)
Supplement: Supplementary file 2 — Additional file 2. Exclusion keywords for journal article literature search. [file 12909_2023_4119_MOESM2_ESM.docx]

Additional file 2: Exclusion keywords for journal article literature search

For each search database listed, Topics 1 through 5 were combined using the term “AND NOT”

Where topic boxes are left blank, adding search terms reduced the yield of papers significantly, such that they were not employed

“ “ denotes exact phrase search

* allows for truncation searching whereby different endings of a word are searched

| **Database** | **Keywords used for exclusion of medical specialties** | | | | |
| --- | --- | --- | --- | --- | --- |
|  | **Exclusion topic 1:**  **Paediatrics** | **Exclusion topic 2:**  **Anaesthetics** | **Exclusion topic 3: Palliative Care** | **Exclusion topic 4: Psychiatry** | **Exclusion topic 5: Obstetrics** |
| **Web of Science** | paediatr* OR pediatr* OR child* | anesthe* OR anaesthe* | palliat* | "psychiatry" | "obstetrics" OR pregnan* |
| **Medline** | paediatr* OR pediatr* OR child* | anesthe* OR anaesthe* | palliat* | "psychiatry" | "obstetrics" OR pregnan* |
| **PubMed** | pregnan*  paediatr* OR pediatr* OR child* | anesthe* OR anaesthe* | palliat* | "psychiatry" | "obstetrics" OR pregnan* |
| **PsychInfo** | paediatr* or pediatr* or child* | anesthe* OR anaesthe* | palliat* | "psychiatry" | "obstetrics" OR pregnan* |
| **ERIC** | Titles manually screened as low numbers/no exclusion function possible | | | | |
| **Open Grey** | Titles manually screened as low numbers/no exclusion function possible | | | | |
| **British library e-thesis online service (EThOS)** | paediatric |  | palliative |  | psychiatry |
