# Supplementary material for: Under- and post-graduate training to manage the acutely unwell patient: a scoping review
Source: BMC Med Educ. 2023 Mar 3;23:146. doi: 10.1186/s12909-023-04119-1 (PMC9983517; doi:10.1186/s12909-023-04119-1)
Supplement: Supplementary file 3 — Additional file 3. Raw Data Set from Scoping Review. [file 12909_2023_4119_MOESM3_ESM.docx]

| **Study number** | **Journal or Abstract** | **Author** | **Title** | **Year published** | **Country** | **Target population** | **Number of participants** | **Descriptive, justification or clarification** | **Theme/theory?** | **Study aim** |
| --- | --- | --- | --- | --- | --- | --- | --- | --- | --- | --- |
| 1 | Journal | Carter M et al | [Didactic lecture versus instructional standardized patient interaction in the surgical clerkship](http://apps.webofknowledge.com/full_record.do?product=UA&search_mode=MarkedList&qid=153&SID=F1nV8MJsasjJWpiatTp&page=1&doc=16&colName=WOS) | 2005 | USA | Medical students | 140 | Justification |  | To investigate whether SPI would increase the self-confidence and be perceived as a more valuable learning tool by surgery students in their history and physical examination (H&P) skills as compared with the classic lecture format |
| 2 | Journal | Beckers S et al | [Evaluation of a new approach to implement structured, evidence-based emergency medical care in undergraduate medical education in Germany](http://apps.webofknowledge.com/full_record.do?product=UA&search_mode=MarkedList&qid=147&SID=F1nV8MJsasjJWpiatTp&page=1&doc=4&colName=WOS) | 2005 | Germany | medical students - 1st year | 236 | Justification |  | Implement structured, evidence-based emergency medical care in undergraduate medical education |
| 3 | Journal | Macdowall | The assessment and treatment of the acutely ill patient--the role of the patient simulator as a teaching tool in the undergraduate programme. | 2006 | UK | medical students - final year | 23 | Justification |  | 1) Improve confidence in management of acutely unwell patient 2) Improve self-assessed ability in management of acutely unwell |
| 4 | Journal | Binstadt, E S. et al | A comprehensive medical simulation education curriculum for emergency medicine residents | 2007 | USA | doctors: emergency medicine residents | All year 1-4 post graduate EM residents at the hospital | Descriptive |  | Create and implement an innovative set of simulation-based modules for integration into current emergency medicine residency curriculum. |
| 5 | Journal | Rettinger, TM | [What effect does an educational intervention have on interns' confidence and knowledge regarding acute dyspnoea management? A randomized controlled trial](http://apps.webofknowledge.com/full_record.do?product=UA&search_mode=MarkedList&qid=153&SID=F1nV8MJsasjJWpiatTp&page=2&doc=70&colName=WOS) | 2006 | USA | interns | 26 | Justification |  | To investigate whether formal case based discussions run by junior doctor (in addition to usual teaching) would improve interns’ knowledge andconfidence in managing patients with acute dyspnea. |
| 6 | Journal | Schwartz LR, et al | A Randomized Comparison Trial of Case-based Learning versus Human PatientSimulation in Medical Student Education | 2007 | USA | medical students - 4th year | 102 | Justification | Case-based learning | Simulation vs case-based learning |
| 7 | Journal | Gruber et al | Teaching acute care: A course for undergraduates | 2007 | Hong Kong | medical students - final year | 155 | Descriptive |  | Describe a course designed to help medical undergraduates develop the necessary competencies to recognise and manage acutely ill patients. |
| 8 | Journal | Shah I, et al | Acute medicine teaching in an undergraduate  medical curriculum: a blended learning approach | 2008 | UK | medical students - final year | 210 | Descriptive |  | To assess impact on confidence using the virtual learning environment (VLE). |
| 9 | Journal | Brunt, LM et al | Accelerated skills preparation and assessment for senior medical students entering surgical internship. | 2008 | USA | Students - final year (4th year) | 31 | Justification |  | Increased preparation to enter surgical residency |
| 10 | Journal | Fuhrmann et al | A multi-professional fulll-scale simulation course in the recognition and management of deteriorating hospital patients | 2009 | Denmark | doctors and nurses (grades not stated) | 220 | Clarification | Experiential learning and adult learning theory | 1) Improve recognition of acutely unwell patient, 2) management of deterioration 3) teamwork/communication |
| 11 | Journal | Carling, J | Are graduate doctors adequately prepared to manage acutely unwell patients? | 2010 | UK | All final yr students took part in programme; 7 F1's followed up for vaildation of programme | Entire year group (no number stated) | Descriptive |  | Improve preparedness in acute illness management |
| 12 | Journal | Schwind CJ et al | [Use of simulated pages to prepare medical students for internship and improve patient safety.](https://www.ncbi.nlm.nih.gov/pubmed/21099392) | 2011 | USA | medical students - 4th year | 16 | Clarification | Activity theory | To pilot the use of simulated pages to improve medical student preparedness, decrease stress related to pages, and familiarize medical students with common patient problems. |
| 13 | Journal | McGlynn M. C. et al | How we equip undergrdauates with prioritsation skills using simuled teaching scenarios | 2012 | UK | medical students - final year | 36 | Descriptive |  | To equip undergrads with skills of 1) task prioritisation, 2) medical management, 3) prescribing and 4) their communication skills with patient and staff through on-call shift where student attends to a set of wards and a bleep. |
| 14 | Journal | Mollo E A | [The Simulated Ward: ideal for Training Clinical Clerks in an Era of Patient Safety](http://apps.webofknowledge.com/full_record.do?product=UA&search_mode=MarkedList&qid=153&SID=F1nV8MJsasjJWpiatTp&page=2&doc=62&colName=WOS) | 2012 | USA | medical students - final year | 89 | Descriptive |  | Gather opinions from surgical clerks reagding novel simulated floor management course to teach patient care concepts required on the surgical wards. |
| 15 | Journal | Wright A et al | [Supporting international medical graduates in rural Australia: a mixed methods evaluation](http://apps.webofknowledge.com/full_record.do?product=UA&search_mode=MarkedList&qid=153&SID=F1nV8MJsasjJWpiatTp&page=2&doc=86&colName=WOS) | 2012 | Australia | Doctors (grades not stated) | 17 | Clarification | Adult learning theory, contextualised theory and reflective practice | To support international medical graduates in rural Australia |
| 16 | Journal | Omrani S et al | [Exploring an Appropriate Instructional Design Model for Continuing Medical Education](http://web.b.ebscohost.com.sheffield.idm.oclc.org/ehost/viewarticle/render?data=dGJyMPPp44rp2%2fdV0%2bnjisfk5Ie46a9It6mxS7Ck63nn5Kx95uXxjL6prUqvpbBIr66eTbips1KxrJ5Zy5zyit%2fk8Xnh6ueH7N%2fiVbKvtVCwqrZLs5zqeezdu33snOJ6u9vwgeGc8nnls79mpNfsVcPAt1G1rrBOpNztiuvX8lXk6%2bqE8tv2jAAA&vid=8&sid=3d7d4613-3021-4d17-b496-3f8a797e1220@sessionmgr120) | 2012 | Iran | General physicans and assistants | 60 | Clarification | Adult learning theory | To compare elearning with traditional learning for continuing education around acute respiratory failure - outcomes knowledge and motivation |
| 17 | Journal | Lovell, B et al | [Simulation training for acute medical specialist trainees: a pilot.](http://ovidsp.tx.ovid.com/sp-3.28.0a/ovidweb.cgi?&S=EFCEFPIPDJDDOECANCFKLGGCCCGFAA00&Complete+Reference=S.sh.71%7c1%7c1) | 2013 | UK | doctors - SPR | 21 | Descriptive |  | Mixed educational and curriculum supporting study: A training day for Acute Medical ST3+s which exposed them to ethically challenging scenarios. The learning objectives were mapped to areas trainees may traditionally describe as either difficult to achieve, or for those for which providing evidence maybe challenging. |
| 18 | Journal | Maddry, JK | A Comparison of Simulation-Based Education versus Lecture-Based Instruction for Toxicology Training in Emergency Medicine Residents | 2014 | USA | doctors: emergency medicine residents | 32 | Justification |  | Our objective was to compare medical simulation (SIM) to traditional lecture-based instruction (LEC) for EM residents in the acute management of critically ill poisoned patients. |
| 19 | Abstract | Rajani, Chandni | The effectiveness of a short HDU placement for foundation Year 1 dcotrs in a district general hospital: A teaching evaluation project | 2014 | UK | F1's | 17 | Justification | learning in and on action | measure outcomes of HDU placement |
| 20 | Abstract | Tuckwell, E et al | Predicting the unpredictable: A pilot study demonstrating the use of simulation techniques in preparing medical students for the on-call shift | 2014 | UK | medical students (final year) | 20 | Descriptive | oncall shift preparation | improve readiness for on-call shifts |
| 21 | Abstract | Hardy, Edward | Novel uses of simulation for students learning the assessment and management of the acutely ill patient | 2014 | UK | medical students (final year) | Not mentioned | Descriptive | combined bedside and trauma/emergency assessment of acutely unwell patient | overcome lack of exposure to acutely unwell patients |
| 22 | Abstract | Eneje, Odiri | CMT SIM: A pilot study using simulation training to prepare core meidcal trainees (CMT) to take on the role of "the Medical Registrar"; trainee's perspectives | 2014 | UK | CMT junior doctors | 6 | Descriptive | transition to registrar | improve confidence to become registrar |
| 23 | Journal | Xu, G et al | [An educational approach to improve outcomes in acute kidney injury (AKI): report of a quality improvement project.](https://www.ncbi.nlm.nih.gov/pubmed/24650804) | 2014 | UK | Doctors - mixed grade | 357 (but not all provided sufficient data for analysis) | Justification |  | Improve awareness of AKI, diagnosis and investigation |
| 24 | Journal | DeWaay, DJ | [Simulation Curriculum Can Improve Medical Student Assessment and Management of Acute Coronary Syndrome During a Clinical Practice Exam](http://apps.webofknowledge.com/full_record.do?product=UA&search_mode=MarkedList&qid=153&SID=F1nV8MJsasjJWpiatTp&page=1&doc=24&colName=WOS) | 2014 | USA | medical students - 4th year | 291 | Clarification | Deliberate pracitce | This study investigated whether a simulation-based curriculum improved a senior medical student's ability to manage acute coronary syndrome as measured during a Clinical Practice Exam, compared to control (no intervention) or didactic teaching. NOTE: simulation group ALSO had didactic teaching. |
| 25 | Abstract | Fadra, Adam et al | A study of high fidelity simulation in pre-clincal to clinical transition in third year medical students | 2015 | UK | medical students (third year) | 35 | Justification | clinical transition | Ease transition from pre-clinical to clinical |
| 26 | Abstract | Broom, Terasa | Does simulation training help to prepare final year medical students for their roles as junor doctors? | 2015 | UK | medical students (final year) | not mentioned | Descriptive | preparation for work | Preparation for practice |
| 27 | Abstract | Hayes, Conrad et al | Simulation-based teaching in using acute ABCDE assessment: improved final year medical student clinical confidence in preparation for foundation years | 2015 | UK | medical students (final year) | 42 | Descriptive | confidence | confidence |
| 28 | Journal | Mughal, Z et al | [Development, Evaluation, and Delivery of an Innovative National Undergraduate Surgical Workshop: Recognition and Management of the Acutely Unwell Surgical Patient](http://apps.webofknowledge.com/full_record.do?product=UA&search_mode=MarkedList&qid=153&SID=F1nV8MJsasjJWpiatTp&page=2&doc=65&colName=WOS) | 2015 | UK | Medical students | 66 | Justification |  | Facilitate the acquisition of knowledge and skill in the early recognition and management of acutely unwell surgical patients. |
| 29 | Journal | Miyasaka, KW, et al | A Simulation Curriculum for Management of Trauma and Surgical Critical Care Patients. | 2015 | USA | doctors: emergency medicine residents | 15 | Justification |  | To reinforce the preparedness and confidence of junior residents in their ability to manage common emergent patient care scenarios in trauma and critical care surgery. |
| 30 | Journal | Dworetzky, B et al | [Interprofessional simulation to improve safety in the epilepsy monitoring unit.](http://ovidsp.tx.ovid.com/sp-3.28.0a/ovidweb.cgi?&S=EFCEFPIPDJDDOECANCFKLGGCCCGFAA00&Complete+Reference=S.sh.69%7c18%7c1) | 2015 | USA | doctors - 2nd year PG (neuro residents ) and neuro nurses | 21 | Clarification | Deliberate pracitce | To train neurology resident–nurse interprofessional teams to maximize effective responses to high-acuity events. |
| 31 | Journal | Christensen, MD et al | [Remotely Versus Locally Facilitated Simulation-based Training in Management of the Deteriorating Patient by Newly Graduated Health Professionals](http://apps.webofknowledge.com/full_record.do?product=UA&search_mode=MarkedList&qid=153&SID=F1nV8MJsasjJWpiatTp&page=1&doc=18&colName=WOS) | 2015 | Australia | nurses and doctors within 12 months of graduation | 305 | Justification |  | This study evaluated delivery of immersive simulation-based training (SBT) by distance education for newly qualified healthcare professionals. |
| 32 | Journal | Cachia, M | [Simulation training for foundation doctors on the management of the acutely ill patient](http://apps.webofknowledge.com/full_record.do?product=UA&search_mode=MarkedList&qid=153&SID=F1nV8MJsasjJWpiatTp&page=1&doc=14&colName=WOS) | 2015 | Malta | foundation doctors | 120 | Descriptive |  | A study evaluating subjective trainee responses to simulation training organized by the Malta Foundation Program in particular whether this changed their clinical practice. |
| 33 | Journal | Stanley, L. et al | A tool to improve competence in the management of emergency patients by rural clinic health workers: a pilot assessment on the Thai-Myanmar border. | 2015 | Thailand | Nurses and doctors | 71 | Justification |  | The development of a tool to improve the competence of local health workers in basic emergency assessment and management |
| 34 | Journal | Arora et al | Crisis Management on Surgical Wards: A Simulation-based Approach to Enhancing Technical, Teamwork, and Patient Interaction Skills | 2015 | UK/USA | doctors first year after qualification | 185 | Justification |  | Using simulation to improve clinical, teamworking and patient-interaction skills for post-op surgical emergencies |
| 35 | Journal | Byrne-Davis L et al | [Efficacy and acceptability of an acute illness management course delivered to staff and students in Uganda by staff from the UK.](http://ovidsp.tx.ovid.com/sp-3.28.0a/ovidweb.cgi?&S=EFCEFPIPDJDDOECANCFKLGGCCCGFAA00&Complete+Reference=S.sh.69%7c20%7c1) | 2015 | Uganda/UK doctors | medical students, student clinical officers, doctors and nurses. | 159 | Justification |  | Increase confidence and knowledge of manaing acutley unwell patients |
| 36 | Abstract | Woods A et al | Inspiring confidence in future doctors : a tailored, near-peer led programme combining theory and simulation teaching for undergraduates | 2016 | UK | medical students | Not mentioned | Clarification | Near-peer | Increase confidence in issues around acutely unwell patient management |
| 37 | Abstract | Boakes, E | Improving the transition from medical student to junior doctor: a one month course in the final year of medical school | 2016 | UK | medical students | Not mentioned | Justification |  | Increase confidence |
| 38 | Journal | MacEwen AW et al | A "Diabetes Acute Care Day" for medical students increases their knowledge and confidence of diabetes care: a pilot study. | 2016 | UK | medical students - 4th year | 272 (144 students completed the pre-course survey and 196 completed the post-course) | Justification |  | To investigate the impact of the introduction of a “Diabetes Acute Care Day” on undergraduate medical students’ knowledge and confidence in acute/inpatient diabetes. |
| 39 | Journal | Cash T et al | [Near-peer medical student simulation training](http://apps.webofknowledge.com/full_record.do?product=UA&search_mode=MarkedList&qid=153&SID=F1nV8MJsasjJWpiatTp&page=1&doc=17&colName=WOS) | 2016 | UK | medical students - 3rd years | 25 | Clarification | Near-Peer learning | To explore if near- peer simulation training is an effective teaching format. |
| 40 | Abstract | Kelly, A | Managing the acutely ill patient upon graduation: A novel, interactive, case-based teaching programme aimed at improving confidence in acute care for final year medical students | 2017 | UK | medical students - final year | 20 | Justification |  | Increase confidence in acute patient management |
| 41 | Abstract | Hoi Ka Wu, C | Transition with Simulation | 2017 | UK | medical students - final year | 21 | Clarification | Contextual learning | To build emotional stamina and for students to remain focused in a stressful situation. |
| 42 | Abstract | Rowland K | Mind the gap: Facilitating the transition between medical student and foundation doctor | 2017 | UK | medical students - final year | around 264 | Descriptive |  | Prepare medical students for the transition into F1 |
| 43 | Abstract | Taylor, S | Transforming the Transition: Medical Student to Junior Doctor | 2017 | UK | medical students - final year | 39 | Descriptive |  | Prepare medical students for the transition into F1 |
| 44 | Journal | Kwan B et al | [Exploring simulation in the internal medicine clerkship](http://apps.webofknowledge.com/full_record.do?product=UA&search_mode=MarkedList&qid=153&SID=F1nV8MJsasjJWpiatTp&page=1&doc=48&colName=WOS) | 2017 | USA | medical students - 3rd years | 43 | Justification |  | Investigators sought to determine the effectiveness of simulation in improving student confidence in acute coronary syndrome (ACS) and the Advanced Cardiac Life Support (ACLS) curriculum. |
| 45 | Journal | Alsaad, A et al | [Assessing the performance and satisfaction of medical residents utilizing standardized patient versus mannequin-simulated training](http://apps.webofknowledge.com/full_record.do?product=UA&search_mode=MarkedList&qid=150&SID=F1nV8MJsasjJWpiatTp&page=1&doc=1&colName=WOS) | 2017 | USA | Residents: internal medicine | 19 | Clarification | Realism | Assess performance and realism of mannikin vs simulated patient in acute scenario management |
| 46 | Journal | McKenzie, S | [Practically prepared? Pre-intern student views following an education package](http://apps.webofknowledge.com/full_record.do?product=UA&search_mode=MarkedList&qid=153&SID=F1nV8MJsasjJWpiatTp&page=2&doc=58&colName=WOS) | 2017 | Australia | medical students - final year | 53 | Clarification | Experiential learning | To examine the impact of a pre-intern (PrInt) education package, consisting of a short intensive course, followed by a one-month clinical attachment. |
| 47 | Journal | Herbstreit, F et al | [Impact of standardized patients on the training of medical students to manage emergencies](http://apps.webofknowledge.com/full_record.do?product=UA&search_mode=MarkedList&qid=153&SID=F1nV8MJsasjJWpiatTp&page=1&doc=41&colName=WOS) | 2017 | Germany | medical students - 4th year | 274 | Justification |  | To compare learning outcomes between traditional seminars vs simulated patients in three emergency clinical scenarios |
| 48 | Abstract | Gan E et al | Preparing medical students for real life practice: a junior resident led OSCE workshop | 2017 | Singapore | medical students - final year | 94 | Descriptive |  | Exposure to common acute scenarios and gaining confdience in managing these |
| 49 | Abstract | Lo, FA et al | Before Taking the Plunge: Preparing our Junior Doctors for the Chaotic Clinical Environment with the Integrated Resuscitation Drill (IRD) | 2017 | Singapore | newly qualified doctors | 40 | Descriptive |  | The Integrated Resuscitation Drill allowed our doctors for hands-on activity in reviving a "collapsed" high-fidelity manikin as part of induction. |
| 50 | Journal | Beane A et al | [Closing the theory to practice gap for newly qualified doctors: evaluation of a peer-delivered practical skills training course for newly qualified doctors in preparation for clinical practice.](http://ovidsp.tx.ovid.com/sp-3.28.0a/ovidweb.cgi?&S=EFCEFPIPDJDDOECANCFKLGGCCCGFAA00&Complete+Reference=S.sh.64%7c18%7c1) | 2017 | Sri Lanka | Pre-interns (newly qualified doctors) | 320 | Clarification | Peer-learning | Improve preparation for clinical pracitce |
| 51 | Journal | Abdeldaim, Y. | Development and implementation of a simulation training program for acute respiratory-failure management in the pulmonary intensive care unit | 2021 | Egypt | Post-graduate residents | 30 | justification |  | Improve knowledge of acute respiratory failure |
| 52 | Journal | AbdelFattah, K. R. | Team-based simulations for new surgeons: Does early and often make a difference? | 2018 | USA | Post-graduate residents | 30 | Clarification | experiential learning | Develop skills related to trauma management, teamwork, and communication. |
| 53 | Journal | Babu, MV | Simulated Patient Environment: A Training Tool for Healthcare Professionals in COVID-19 Era | 2021 | India | Doctors, epidemiologists, nurses, laboratory technicians, laboratory attendants, members of infection control team and biomedical waste management team | 50 | justification |  | Improve the preparedness and control measures to be taken for respiratory Infection Outbreak in a hospital |
| 54 | Journal | Bongers et ak | Cross-Cover Curriculum for Senior Medical Students | 2020 | USA | medical students (final year) | 29 | Justification |  | Improve preparedness and clinical decision making for 'cross cover' (out of hours cover) |
| 55 | Journal | Brailovsky et al | Pulmonary embolism response team implementation improves awareness and education among the house staff and faculty | 2020 | USA | residents, junior and senior faculty | 115 | Justification |  | To improve PERT awareness, familiarity with treatment options, role of echocardiogram and Doppler ultrasound, and knowledge of acute PE risk stratification tools. |
| 56 | Journal | Cetrone, E. | Training junior doctors to lead rapid responses | 2021 | USA | PG Y1 doctors | 48 | Clarification | Transition Theory (Schlossberg) | Increase preparedness to lead rapid response sceenarios |
| 57 | Journal | Church et al | Using Insights From Sports Psychology to Improve Recently Qualified Doctors’ Self-Efficacy While Managing Acutely Unwell Patients | 2021 | UK | PG years 1 and 2doctors | 12 | Clarification | Metacognition | Improve negative emotions and behaviours during management of acutely unwell patients using sport psychology techniques |
| 58 | Journal | Dave et al | Improving knowledge and confidence in foundation doctors during specialty changeover | 2020 | UK | PG year 1 | 37 pre-session, 23 post-session | Clarification | Scaffolding? | Improve knowledge of specialty pre-changeover |
| 59 | Journal | Drost-de Klerck, A. M. | Use of simulation training to teach the ABCDE primary assessment: an observational study in a Dutch University Hospital with a 3-4 months follow-up | 2020 | Netherlands | PG year 1 | 30 | Justification |  | To investigate short-term and long-term effectiveness of simulation training to acquire a structured Airway Breathing Circulation Disability Exposure (ABCDE) approach for medical emergencies; and t |
| 60 | Journal | Gallagher,K et al | Successful prospective quality improvement programme for the identification and management of patients at risk of sepsis in hospital | 2019 | UK | Foundation doctors | Not specified as patient data was the outcome measure | Justification |  | This audit aimed to improve the speed and completeness of delivery of treatment to urology patients at risk of sepsis in the hospital.antibiotic |
| 61 | Journal | Jeimy et al | Evaluation of virtual patient cases for teaching diagnostic and management skills in internal medicine: a mixed methods study | 2018 | Canada | Medical students and residents | 52 | Justification |  | Does use of Virtual Patient improve knowledge around common medical problems compared to powerpoint presentation lecture |
| 62 | Journal | Kulshreshtha, P.et al | Preparedness of Undergraduate Medical Students to Combat COVID-19: A Tertiary Care Experience on the Effectiveness and Efficiency of a Training Program and Future Prospects | 2022 | India | final and pre-final year medical students | 179 | Justification |  | to analyze the effectiveness and efficiency of preparedness training to combat COVID-19 in pre-final and final-year medical students |
| 63 | Journal | Lammers, R et al | A Simulated Case of Acute Salicylate Toxicity From an Intentional Overdose | 2018 | USA | 2nd year medical students | 60 | Descriptive |  | (1) demonstrate the symptoms and signs of salicylate toxicity, (2) provide practice indiagnosing a mixed acid-base disturbance, and (3) introduce students to various treatment modalities forthis poisoning. |
| 64 | Journal | Mallik et al | Exploring the Role of Virtual Reality to Support Clinical Diabetes Training-A Pilot Study | 2022 | UK | PG doctors (trainees) and Trainee ACPs | 39 | Descriptive |  | to evaluate the use of virtual reality to help non-specialist clinicians manage clinical scenarios related to diabetes |
| 65 | Journal | Malmut, L et al | Simulation-Based Education for Urgent Medical Complications Commontothe Rehabilitation Setting: An Educational Program for Physical Medicine and Rehabilitation Residents | 2019 | USA | PG year 2 doctors | 12 | Clarification | Retrieval practice and Experiential Learning | To examine whether a simulation-based educational program can improve PM&R resident confidence and knowledge in the assessment and management of urgent medical complications |
| 66 | Journal | Patel, Rima et al | An Interactive Mock Paging Curriculum to Prepare New Internal Medicine Interns for Inpatient Wards | 2021 | USA | Interns | 20 | Clarification | Problem-based learning | To improve knowledge, comfort, and skillsin managing routine inpatient encounters |
| 67 | Journal | Rabbabah et al | Effectiveness of an educational program on improving healthcare providers’ knowledge of acute stroke: A randomized block design study | 2021 | Jordan | Physcians, nurses and paradmedics | 189 | Justification |  | Improve knowledge of stroke management and access to protocols |
| 68 | Journal | Solanki | Use of high-fidelity simulated cases to improve thirdyear medical students’ ability to manage an acutely unwell patien | 2017 | UK | 3rd year medical students | 52 | Clarification | Experiential learning | Opportunity to treat sick patients, and measure any improvement in knowledge |
| 69 | Journal | Wallet et al | Developing a simulation-based learning model for acute medical education during COVID-19 pandemic with Simulation via Instant Messaging – Birmingham Advance (SIMBA) | 2022 | UK | FY1, SHOs, Registrars | 75 | Clarification | Experiential learning | (i) develop an SBL tool to improve case management; (ii) evaluate experiences and confidence before and after; and (iii) compare efficacy across training levels. |
| 70 | Abstract | Phillips et al | Does Multi-disciplinary debriefing improve clinical knowledge, human factors and confidence in interprofessional simulation | 2019 | UK | nursing and medical students | 10 | Clarification | Interprofessional education, scaffolding | Investigate whether debriefing with nurses and doctors, compared to doctors alone, improved knowledge an confidence in acute management |
| 71 | Abstract | Murphy et al | Preparing final year medical students for clinical practice by using simulated teaching scenarios | 2020 | UK | medical students | 197 | Descriptive |  | Preparation for practice |
| 72 | Abstract | Fry et al | EscapiSIM | 2020 | UK | medical students | Not stated | Descriptive |  | Improve working under pressure |
| 73 | Abstract | Thompson et al | Near--peer surgical simulation and teaching day for FY1 doctors | 2020 | UK | FY1 doctors | 44 | Clarification | Near-peer | Preparation for practice |
